# Automatic Grade Classification in Prostate Histopathological Images Using EfficientNet and Ordinal Focal Loss

**DOI:** 10.3390/bioengineering13050503

**Published:** 2026-04-26

**Authors:** Woshington Valdeci de Sousa Rodrigues, Armando Luz, José Denes Lima Araújo, João Diniz, Antonio Oseas Filho

**Affiliations:** 1 Instituto Federal do Piauí (IFPI), Campus Picos, Picos 64605-500, PI, Brazil; 2 Universidade Federal do Piauí (UFPI), Campus Picos, Picos 64607-760, PI, Brazil; armandoluzborges@ufpi.edu.br (A.L.); jose.denes@ufpi.edu.br (J.D.L.A.); antoniooseas@ufpi.edu.br (A.O.F.); 3 Fábrica de Inovação, Instituto Federal do Maranhão–Campus Grajaú, Grajaú 65940-000, MA, Brazil; joao.bandeira@ifma.edu.br

**Keywords:** prostate cancer, histopathological image classification, EfficientNet, ordinal regression, focal loss, digital pathology, PANDA dataset

## Abstract

The automatic classification of ISUP (International Society of Urological Pathology) grade groups in prostate histopathological images remains challenging due to the high similarity between adjacent classes, class imbalance, and label noise. In this work, we propose a deep learning pipeline based on EfficientNet convolutional neural networks combined with a hybrid loss function that integrates ordinal regression and Focal Loss to better capture the ordered nature of ISUP grades. A noise-filtering strategy based on the entropy of predictions from multiple EfficientNet models was first applied to identify and remove high-uncertainty samples from the training set. The problem was then reformulated as an ordinal regression task to explicitly model the hierarchical relationship among grades. Experiments conducted on the PANDA dataset demonstrate that removing noisy samples improved performance from κ=0.826 to κ=0.833. Incorporating ordinal loss further increased performance to κ=0.851. The best configuration, combining ordinal regression and Focal Loss, achieved κ=0.857 and an accuracy of 0.669, while reducing severe misclassifications and concentrating errors among adjacent classes. These results indicate that explicitly modeling ordinal structure and mitigating label noise are effective strategies for improving prostate cancer grading systems.

## 1. Introduction

Prostate cancer is one of the most common malignant neoplasms in men, representing a significant public health challenge [1]. Statistics reported by the International Agency for Research on Cancer indicate that in 2022 this neoplasm was the fourth most common worldwide, accounting for 7.3% of all cancer cases [2]. In this context, tumor grading plays a central role in determining clinical management and prognosis. The Gleason system, based on the sum of the two predominant histological patterns, evolved into the grading system proposed by the International Society of Urological Pathology (ISUP), organized into five groups ranging from 1 to 5. This system provides improved prognostic stratification and is widely adopted in clinical practice [3].

The traditional histopathological evaluation of Whole-Slide Images (WSIs) relies on manual analysis performed by pathologists. Given the large number of fields that must be examined within a single slide, the evaluation process becomes a repetitive task that requires long working hours, sustained attention, and specialized expertise. Fatigue resulting from prolonged analysis may introduce inter- and intra-observer variability in results, directly influencing patient clinical management [4]. Consequently, in recent years, there has been significant growth in the use of Artificial Intelligence (AI) techniques to automate the analysis of histopathological examinations and assist in prostate cancer diagnosis, aiming to mitigate the aforementioned limitations [5].

In this scenario, Convolutional Neural Networks (CNNs) have represented the state of the art in medical image analysis [6], being capable of extracting relevant features and combining complex visual patterns to identify specific pathological conditions [7]. This capability makes the approach promising for the automated analysis of Gleason patterns and ISUP grade groups, thereby reducing the variability inherent to conventional evaluation. Based on this premise, deep learning techniques for prostate grading have been widely investigated in the literature, with notable advances in WSIs, patch extraction strategies, and multi-scale aggregation mechanisms. For example, in the context of weakly supervised learning, ref. [8] proposed the GCN-MIL framework, which uses a Graph Neural Network to aggregate spatial features of patches on WSI slides. The model incorporates a Robust Training strategy that iteratively filters high-uncertainty samples to mitigate the impact of noisy labels, achieving a quadratic kappa of 0.931 on the internal PANDA challenge dataset. However, the approach employs the traditional Cross-Entropy loss function, which treats ISUP grades as independent nominal classes and ignores the biological hierarchy of cancer progression.

In an independent clinical validation with 593 images from the *Seoul National University Hospital*, ref. [9] evaluated the DeepDx Prostate system, achieving a quadratic kappa of 0.922 in Gleason grading. However, the model’s generalization was limited by the homogeneity of the institutional dataset and by a tendency to overestimate Grade Group 1 (GG1) cases. Seeking indirect risk prediction, ref. [10] investigated the use of deep learning to identify patients at risk of cancer from slides previously classified as benign. The method achieved an AUC of 0.739 for risk prediction, revealing subtle morphological patterns, but was limited by reliance on a single institutional domain.

To address the inherent scale variability of glandular structures, ref. [11] proposed a *Pyramidal CNN* architecture composed of three shallow networks operating on different patch sizes. Although the method achieved approximately 0.77 accuracy in pattern classification, it was limited by semi-automatic labeling and moderate performance in more complex classes. In parallel, aiming to improve interpretability, ref. [12] evaluated architectures combined with *Explainable AI* techniques (*Grad-CAM*) using the SICAPv2 dataset. The VGG19 model achieved the best performance (AUC-ROC of 0.85), although the study lacked external clinical validation. Following a simplification strategy, ref. [13] formulated the detection problem as a binary classification task (benign vs. malignant tissues). Using *fine-tuning* on InceptionResNetV2 models with the *binary cross-entropy* loss, they achieved 91.8% accuracy, emphasizing detection over multiclass Gleason grading.

In advanced architectures, the incorporation of attention mechanisms has also gained prominence. Ref. [14] proposed a deep learning model based on the EfficientNet-B4 architecture with a channel attention mechanism (Eff4-Attn), achieving 93.32% accuracy in an eight-class classification task using images from the *DiagSet*. However, the study relies exclusively on isolated patches and suffers from class imbalance. Complementarily, ref. [15] investigated the use of *Vision Transformers* (ViT) on a subset of the PANDA dataset, achieving patch classification accuracy above 85%, highlighting the ability of attention mechanisms to capture global contextual dependencies.

More recent studies have explored advanced strategies to improve robustness and clinical alignment in prostate cancer grading. Ref. [16] proposed a hierarchical ensemble of convolutional neural networks, combining global and specialized models to resolve confusion between similar ISUP grades, achieving a quadratic kappa of 0.881 on the PANDA dataset. Similarly, ref. [17] introduced a three-stage framework integrating patch-level classification and semantic segmentation, followed by machine learning classifiers based on Gleason pattern proportions, reaching a kappa of 0.921.

Focusing on the ordinal nature of the problem, ref. [18] proposed the TSOR framework, which combines multiple instance learning, self-supervised learning, and ordinal regression to capture hierarchical relationships among tumor grades better, achieving competitive performance on both the PANDA and SICAPv2 datasets.

Despite recent advances, several methodological gaps remain. Many studies treat the problem as a nominal classification task and employ traditional loss functions, disregarding the ordinal nature of ISUP grades. Consequently, prediction errors are penalized uniformly, without accounting for the magnitude of the discrepancy between classes. Moreover, many approaches do not incorporate robust mechanisms for noise filtering or uncertainty modeling, an important aspect given the presence of artifacts and inconsistencies in public datasets.

Although some recent work has explored ordinal regression strategies, these approaches are still not widely adopted and are often not combined with robust noise-handling mechanisms.

To address these limitations, this work proposes a pipeline for the automatic classification of ISUP grade groups in histopathological images. The main contributions are: (i) a hybrid loss function that combines ordinal penalization and focal loss, considering the hierarchical nature of the problem and class imbalance; (ii) a noise removal strategy based on uncertainty estimated from the average predictions of EfficientNet models (B0, B1, B2, and B3); and (iii) a comparative evaluation of EfficientNet architectures B0, B3, and B7, aiming to obtain a more robust and consistent inference model.

## 2. Materials and Methods

**Figure 1:**
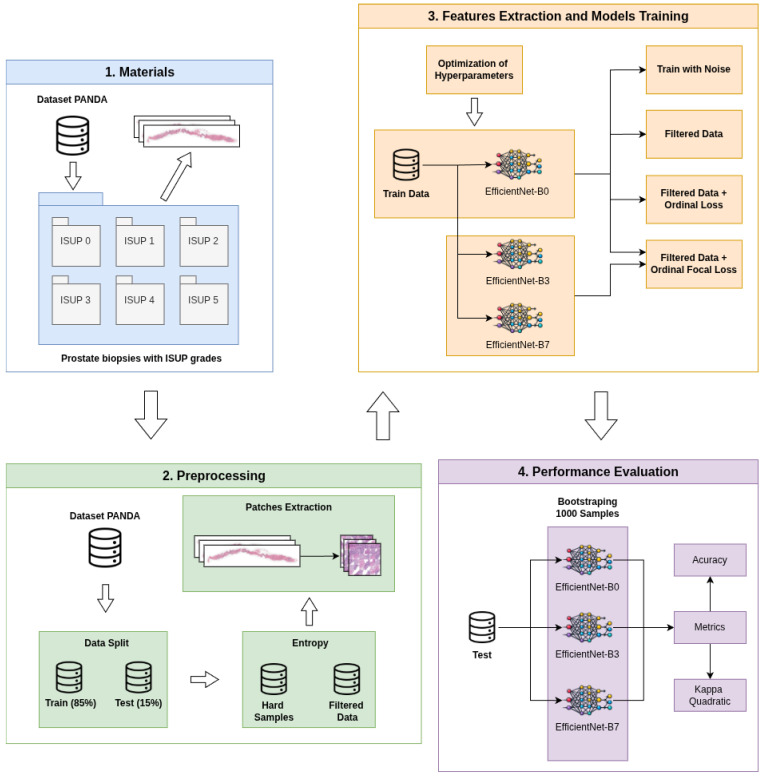
Proposed method.

This section presents the methodological procedures used to develop and evaluate the proposed approach. The method, illustrated in Figure 1, is structured in four main stages: (i) materials, (ii) preprocessing, (iii) feature extraction and model training, and (iv) performance evaluation. Each stage is described in detail in the subsections below.

### 2.1. Materials

In this study, the PANDA (*Prostate Cancer Grade Assessment*) dataset was used, which consists of approximately 10,616 whole-slide histopathological images of prostate biopsies [19]. This dataset was provided as part of the PANDA challenge on the Kaggle platform and has been widely used in research on automatic prostate cancer grading.

The images in the dataset are annotated with Gleason scores and Gleason grades, providing clinical labels used for disease grading. In this study, only the public version of the dataset was used, without access to the private test set provided during the challenge.

The data were collected from two medical centers, *Radboud University Medical Center* and *Karolinska Institute*, introducing institutional variability in the images. This variability is associated with differences in slide preparation protocols, scanning procedures, and annotation criteria, thereby making the experimental setting more challenging.

**Figure 2:**
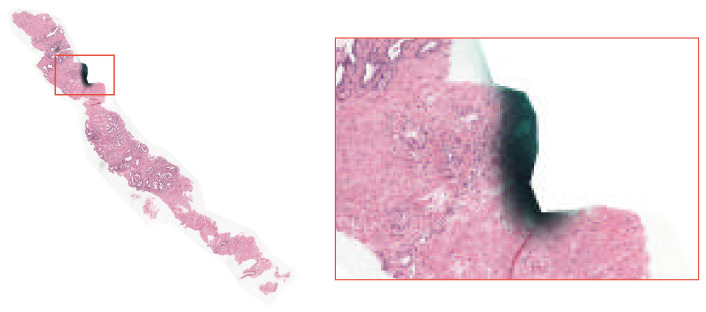
Example of a visual artifact present in a WSI from the PANDA dataset. On the left, the whole slide is shown with the region of interest highlighted in red. On the right, a zoomed-in view of the selected region reveals a pen mark over the prostate tissue.

Additionally, the dataset contains inherent noise from the slide acquisition and annotation processes. Visual artifacts include pen marks made by pathologists during slide analysis, as illustrated in Figure 2. There are also noise sources related to labeling, such as disagreement among annotators in assigning histopathological grades. These factors increase the complexity of the automatic classification task and reflect challenges present in digital pathology practice.

**Figure 3:**
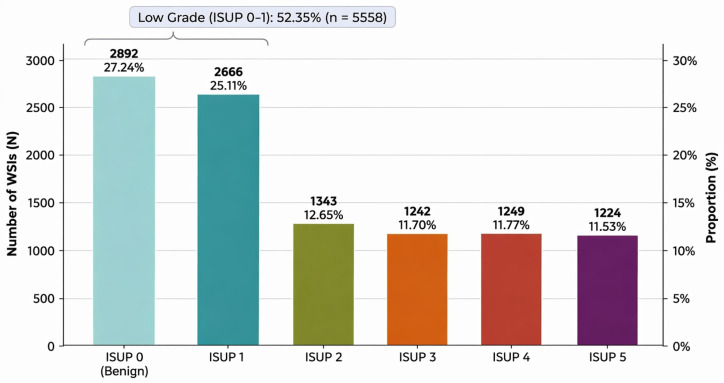
Sample distribution in the PANDA dataset.

Another relevant aspect of the dataset is the uneven distribution of samples across the six grading classes (ISUP 0 to ISUP 5). This imbalance is evident in Figure 3, which presents the distribution of samples per class. Most examples are concentrated in the ISUP 0 and ISUP 1 classes.

### 2.2. Preprocessing

The preprocessing workflow consisted of two sequential stages: label-noise filtering and patch extraction. Initially, the dataset was split into training (85%) and test (15%), ensuring independence between model fitting and final evaluation. After this split, the training set contained 9024 instances, while the test set, used for evaluating all models in this study, consisted of 1592 instances.

#### 2.2.1. Entropy-Based Noise Filtering

As reported in Section 2.1, the PANDA dataset contains label noise due to manual annotations and inconclusive pathological reports. To mitigate its impact, a filtering strategy based on predictive uncertainty estimation was adopted. Initially, four low-complexity variants of the EfficientNet family (B0, B1, B2, and B3) were trained, reserving 20% of the training set for internal validation. Each model then produced probability outputs for all training and validation samples.

Given that the network outputs independent probabilities per node via a sigmoid activation, predictive uncertainty was quantified using Shannon entropy computed directly from these outputs, without explicit class assignment [20]. Considering *C* as the number of output nodes and $p_{c}$ as the predicted probability for the *c*-th node, the mean binary entropy for a sample is defined as:

(1) $$H=\frac{1}{C}\sum_{c=1}^{C}-\left[p_{c}\log\left(p_{c}\right)+\left(1-p_{c}\right)\log\left(1-p_{c}\right)\right]$$

Predictions with probabilities closer to 0 or 1 yield lower entropy (higher confidence), whereas values near 0.5 indicate higher uncertainty. Entropy was computed per sample for each model and then averaged across the four networks. The top 10% highest-entropy samples were selected as *hard samples*. Their removal from the training set was evaluated under four scenarios: (i) no removal and (ii) removal of 100%, (iii) 50%, and (iv) 20% of the *hard samples*.

#### 2.2.2. Patch Extraction

**Figure 4:**
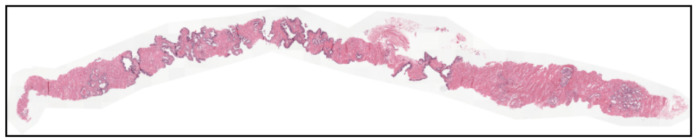
Example image from the PANDA dataset showing a large tissue-free area.

After defining the training samples through entropy filtering, the final preprocessing stage consisted of generating input images for the final model. Since the histological tissue occupies only a fraction of the WSI, as shown in Figure 4, processing the full slide would require a high and unnecessary computational cost due to extensive background regions.

To extract informative regions from the images, a patch-based approach was adopted. A fixed patch size of 256×256 pixels was defined. To ensure standardization, white padding was applied to the WSI borders, guaranteeing exact divisibility into patches of this size. Then, each image was partitioned, and tissue presence was estimated by summing the RGB values of each patch; since the background tends to be white, patches with lower sums indicate higher tissue density. The patches were then sorted in ascending order according to this metric. In cases where the WSI contained insufficient tissue to reach the desired number of patches, the remaining positions were filled with white patches.

**Figure 5:**
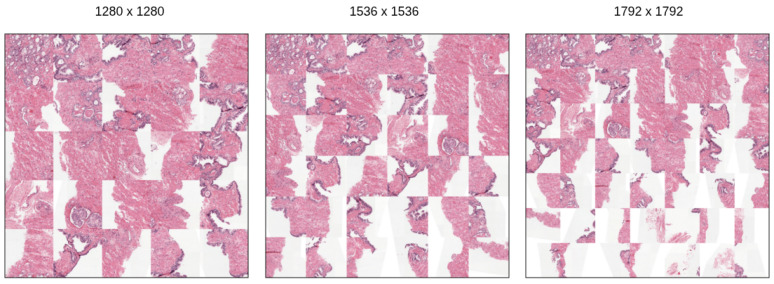
Illustration of patch extraction at different sizes: 5 × 5 (**left**), 6 × 6 (**center**), and 7 × 7 (**right**).

Finally, the spatial arrangement of the patches was evaluated in 5 × 5, 6 × 6, and 7 × 7 configurations (Figure 5). The 5 × 5 configuration omitted subtle lesion patterns, while the 7 × 7 configuration generated high-dimensional images (1792 × 1792) with excessive background. The intermediate 6 × 6 configuration (36 patches, producing 1536 × 1536 images) was adopted to balance preservation of tissue information, spatial coverage, and computational efficiency.

### 2.3. Feature Extraction and Model Training

This section describes the strategy for training the classifiers, following the image preprocessing stage. Initially, the EfficientNet-B0 architecture was adopted as the base model for hyperparameter tuning due to its balance between representational capacity and computational efficiency. Once the optimal configuration was defined, experiments were extended to the EfficientNet-B3 and EfficientNet-B7 architectures. This methodological progression enabled the assessment of the impact of increased structural complexity on model performance and generalization.

During hyperparameter evaluation and baseline model construction, the BCE loss function served as the initial reference. Different combinations of learning rate, dropout, and proportion of unfrozen layers for *fine-tuning* were investigated through a grid search using the EfficientNet-B0 architecture. Even after this optimization, framing the problem as conventional multiclass classification limited the model’s performance. Considering that ISUP grades represent an ordinal progression of tumor severity, the problem was reformulated as an ordinal regression task to capture the hierarchical dependence among classes.

In this approach, ISUP grade prediction is not treated as a multiclass problem with independent categories, but rather as a sequence of cumulative binary classifications. For an ISUP grade k∈{0,…,5}, the label is encoded as a cumulative binary vector where the first *k* elements are set to 1 and the remaining elements to 0. For example, grade 3 is represented as [1,1,1,0,0]. This encoding enables the model to learn progressive thresholds associated with increasing disease severity.

To exploit this ordinal structure, a composite loss function combining two complementary terms was employed. The first term corresponds to Binary Cross-Entropy (BCE) applied to the cumulative thresholds. To address class imbalance and emphasize hard samples, this term is modulated using the focal loss mechanism [21]. Considering ${\hat{p}}_{i}=\sigma \left(z_{i}\right)$ as the predicted probability for threshold *i*, where σ(·) denotes the sigmoid function and $z_{i}$ represents the corresponding logit, the focal loss is defined as:

$$L_{focal}=-\frac{1}{K}\sum_{i=1}^{K}\alpha_{t}{\left(1-p_{t}\right)}^{\gamma }\log\left(p_{t}\right)$$

where

$$p_{t}={\hat{p}}_{i}y_{i}+\left(1-{\hat{p}}_{i}\right)\left(1-y_{i}\right)$$

and

$$\alpha_{t}=\alpha y_{i}+\left(1-\alpha \right)\left(1-y_{i}\right)$$

Here, $y_{i}$ denotes the binary target associated with threshold *i*, γ is the focusing parameter that reduces the contribution of easy samples, and α is a balancing factor between classes.

The second term introduces a penalty based on the absolute difference between the model’s predicted class and the true label. The expected class is obtained by summing the probabilities associated with the predicted thresholds.

Formally, the estimated class is defined as:

$$\hat{y}=\sum_{i=1}^{K}{\hat{p}}_{i}$$

where *K* is the number of ordinal thresholds. The ordinal penalty is then given by:

$$|\hat{y}-y|$$

where *y* represents the ground truth class.

The final loss function is defined as:

$$L=\lambda \cdot L_{focal}+\beta \cdot \left|\hat{y}-y\right|$$

where λ and β control the relative contribution of the classification and ordinal penalty terms, respectively. This formulation preserves the ordinal structure of the problem by penalizing more severe misclassifications, while simultaneously emphasizing difficult samples through the focal mechanism.

### 2.4. Performance Evaluation

After defining the methodological pipeline, encompassing patch extraction, hyperparameter optimization, and loss function adjustment, the performance of the EfficientNet architectures (B0, B3, and B7) was evaluated on unseen data.

#### Metrics and Statistical Analysis

Model predictive performance was measured using Accuracy, Precision, F1-score, and Quadratic Weighted Kappa. Accuracy reflects the overall proportion of correct predictions [22]. Precision (macro-averaged) measures the proportion of correctly predicted samples among all samples predicted for each class, providing insight into the reliability of positive predictions across classes [23]. The F1-score (macro-averaged), defined as the harmonic mean of precision and recall, balances false positives and false negatives, offering a more comprehensive evaluation in the presence of class imbalance [24].

The kappa quadratic metric assesses agreement between predictions and true labels while applying penalties proportional to the magnitude of errors. This property makes it particularly suitable for handling the ordinal progression inherent in ISUP grading [25].

To quantify uncertainty and variability in the results, a non-parametric *bootstrap* method was applied [26]. A total of 1000 resamplings with replacement were generated from the test set predictions, preserving the original sample size. In each iteration, the metrics were recalculated to estimate the mean, standard deviation, and 90% confidence interval (percentiles 5% and 95%). This strategy ensures a robust statistical assessment, independent of assumptions about the underlying data distribution.

## 3. Results

This section presents the experimental results obtained from applying the methodology described in Section 2. The objective is to validate the proposed approaches and analyze the model’s behavior under varying conditions. Initially, the computational environment and the hyperparameters used to train the baseline model are described. Next, the impact of the noise-removal stage and different loss-function formulations on predictive performance is investigated. Finally, a comprehensive comparative study across different EfficientNet architectures is conducted, evaluating the trade-off between computational cost and effectiveness.

### 3.1. Experimental Environment

The experiments were conducted on a Linux workstation equipped with an AMD Ryzen 5 5600X processor (Advanced Micro Devices, Santa Clara, CA, USA), 32 GB of DDR4 RAM (3200 MHz), and an NVIDIA GeForce RTX 3060 GPU (NVIDIA Corporation, Santa Clara, CA, USA) (12 GB of VRAM), dedicated to accelerating the training of deep learning models. The development was carried out in Python 3.10.0 the using the PyTorch 2.5.1 framework integrated with CUDA 12.8. For histopathological image processing, the OpenSlide library 1.4.3 was used to read images and extract patches. The evaluation metrics were computed using scikit-learn (version 1.4.2), and the visual analyses were generated with Matplotlib (version 3.8.4).

### 3.2. Hyperparameter Configuration

The initial stage of the experimentation consisted of a grid search to identify the hyperparameter configuration that maximized convergence and generalization of the baseline model, thereby mitigating the risk of overfitting. The explored values and the final selected configuration are summarized in Table 1. This optimal configuration was established as the standard and kept constant in subsequent experiments to ensure comparability of the results.

The initial learning rate set to $3\times 10^{-4}$, operating together with the Adam optimizer, provided the best stability during the minimization of the loss function (BCE). For fine-tuning, unfreezing the last 150 layers of the network yielded the best balance. In addition, a dropout layer with a retention probability of 0.4 proved to be the most effective regularization strategy. The batch size was limited to two samples due to GPU memory constraints. To mitigate fluctuations in gradient updates due to the small batch size, training was configured for up to 50 epochs, using an early stopping criterion with a patience of 7 epochs after no improvement in the validation metric.

To improve model generalization and increase robustness to variability in data acquisition conditions, data augmentation was applied on-the-fly during training using the Albumentations library. The adopted transformations include random transpositions, horizontal and vertical flips, which increase the variability of the training samples and help mitigate overfitting.

Although the PANDA dataset contains a substantial number of whole-slide images, the main limitation lies in the limited institutional variability, as data from both institutions are used during training. Therefore, data augmentation plays an important role in improving robustness to potential domain shifts.

### 3.3. Impact of Noise Filtering and Loss Functions

With the optimal set of hyperparameters defined, the baseline model was trained using the EfficientNet-B0 architecture and the BCE loss function. This model achieved an accuracy of 0.592 and a Kappa coefficient of 0.826. In the context of ISUP grade classification in prostate biopsies, this Kappa value is considered highly satisfactory and competitive. The medical literature reports considerable interobserver variability in histopathological grading [27,28]. While experienced uropathologists often achieve Kappa values between 0.43 and 0.68 [27], more recent evaluations specifically focused on ISUP grades report minimal agreement levels among pathologists, with Kappa values around 0.31 [28]. Therefore, the obtained baseline demonstrates agreement that considerably exceeds the reported human variability. Furthermore, the results indicate that even with a moderate accuracy (59.2%), most prediction errors are concentrated between adjacent ISUP grades.

The entropy-based filtering stage identified a subset of 903 samples, corresponding to the 10% with the highest uncertainty among the predictions of EfficientNet-B0, B1, B2, and B3 models. The analysis of this subset revealed characteristics indicating great difficulty for the models, including high mean entropy (0.3851±0.0638), high variability in predictions (1.1188±0.8943), and low agreement among the evaluated architectures. It was observed that only 6.2% of the samples presented consensus among the four models. Additionally, in 58% of instances classified as *hard samples*, none of the models produced the correct prediction. These findings support the hypothesis that entropy can act as an effective indicator for identifying difficult or inconsistent examples.

**Figure 6:**
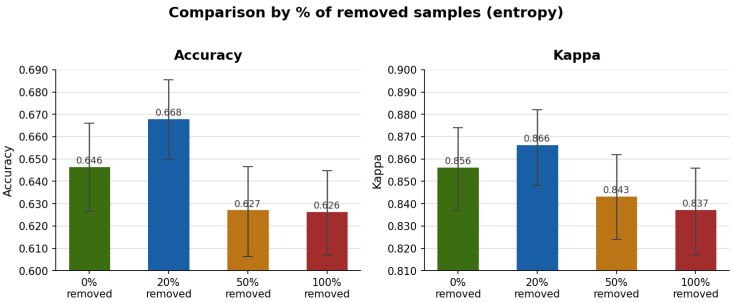
Performance under different noise removal thresholds applied to the *hard samples*.

Based on this subset of *hard samples*, different scenarios for removing potentially noisy data during training were investigated. The results indicated that partial removal of these samples contributed to improved model performance. As shown in Figure 6, removing approximately 20% of the *hard samples* yielded the best balance between noise reduction and preservation of the training set’s diversity, suggesting that moderate filtering may improve the model’s generalization capability.

**Figure 7:**
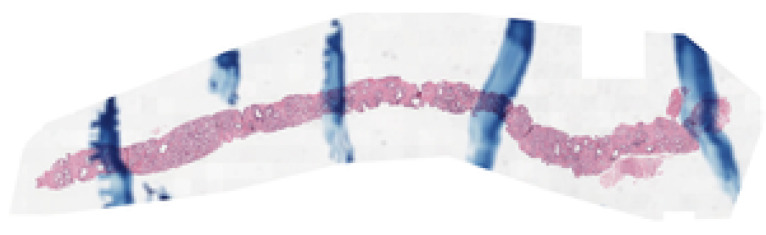
Image identified as noise by the filtering method.

Figure 7 presents an example of an image identified by the method as a sample with high prediction uncertainty. When analyzing the models’ predictions, the true label is ISUP class 2. However, during inference, EfficientNet-B0 classified it as ISUP 3, whereas EfficientNet-B1, B2, and B3 predicted ISUP 1. Although these discrepancies do not constitute clinically severe errors, the analysis at this stage prioritizes the uncertainty associated with model decisions. For this specific image, the calculated mean entropy value was 0.59, which is considered high. This example illustrates how the method can identify potentially noisy cases; in this particular image, pen annotations are clearly visible. The effectiveness of this filtering strategy becomes more evident when analyzing the results presented in Table 2. The application of noise removal increased $\kappa_{\mathrm{quad}}$ from 0.826 in the baseline model to 0.833. The impact on accuracy was even more pronounced, increasing from 0.592 to 0.638, corresponding to an absolute gain of approximately 4.6 percentage points. These results indicate that removing a fraction of highly uncertain samples improves the model’s generalization capability.

**Figure 8:**
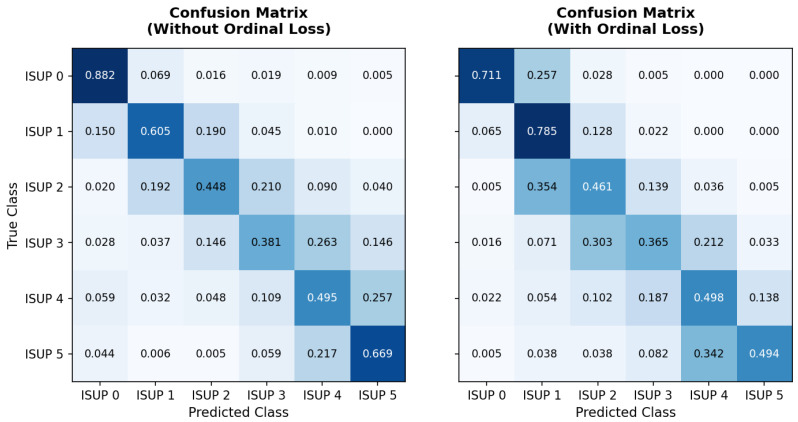
Normalized confusion matrices obtained for the model trained with BCE loss (**left**) and ordinal loss (**right**). The color intensity (shades of blue) represents the proportion of samples, with darker shades indicating higher values.

Replacing the BCE loss function with the ordinal formulation increased the overall model performance to 0.851, although a slight reduction in accuracy was observed. This result suggests that incorporating the ordinal factor redistributes incorrect predictions, reducing errors between distant classes and concentrating confusions among adjacent classes. In ordinal classification problems, such as the ISUP grading system, this behavior is desirable because discrepancies between neighboring classes have less clinical impact than errors between distant grades. This behavior is shown in Figure 8, which presents normalized confusion matrices for models trained with BCE and the ordinal loss function. It can be seen that the model trained with an ordinal loss concentrates errors primarily between adjacent classes, whereas the BCE-trained model exhibits greater dispersion toward more distant classes.

Analyzing the main diagonal of the matrices reveals improvements in ISUP classes 1, 2, and 4, whereas reductions in accuracy were observed for ISUP classes 0, 3, and 5. Moreover, the ordinal loss almost eliminates errors between distant classes. Samples classified as ISUP 0 are no longer classified as ISUP 3, 4, or 5 and are predominantly misclassified as the adjacent class ISUP 1. Conversely, greater dispersion is observed in intermediate classes, particularly ISUP 2 and ISUP 3, reflecting the intrinsic difficulty of distinguishing adjacent histopathological grades. Overall, the results indicate that the use of ordinal loss promotes classification behavior more aligned with the hierarchical nature of the problem, reducing severe discrepancies between classes and favoring confusions between adjacent histopathological grades.

After introducing the ordinal loss, it was observed that dataset imbalance, described in Section 2.1, could be limiting model performance. Therefore, a hybrid loss function that combines ordinal penalization with the focal mechanism was proposed to emphasize more difficult samples and mitigate the effects of class imbalance.

This strategy achieved the best overall performance among the evaluated configurations, reaching a quadratic kappa of 0.857 and an accuracy of 0.669. In addition, it consistently improved classification quality across complementary metrics, achieving a macro F1 Score of 0.612 and macro precision of 0.628 (Table 2).

**Figure 9:**
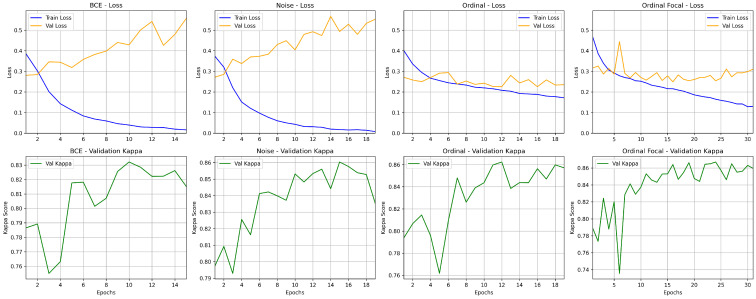
Loss and kappa curves during training and validation for different strategies.

The learning dynamics illustrated in Figure 9 provide important insights into the behavior of each training strategy. The baseline model (BCE) exhibits clear overfitting: while the training loss decreases rapidly, the validation loss steadily increases after the first epochs. This divergence is reflected in the validation QWKappa, which improves initially but then fluctuates and stabilizes at a lower level, indicating limited generalization.

The application of entropy-based noise removal partially mitigates this issue. Although the validation loss still shows an increasing trend, its rate of increase is less abrupt than the baseline. This results in a more stable improvement in QWKappa, which reaches higher values than BCE, suggesting that removing highly uncertain samples improves the quality of the training signal, even if overfitting is not fully eliminated.

The introduction of the Ordinal Loss leads to a noticeable change in training dynamics. Both training and validation losses become more stable, with a smaller gap between them, indicating better generalization. This is reflected in the smoother, more consistent increase in QWKappa, which reaches competitive values while exhibiting fewer fluctuations than previous approaches.

Finally, the Hybrid approach (Ordinal + Focal) demonstrates the most stable convergence behavior. The validation loss remains relatively stable throughout training, despite some oscillations, and the gap between the training and validation losses is smaller than with BCE-based methods. The QWKappa curve shows a consistent upward trend with reduced variability, achieving the highest and most stable performance among all configurations. These results suggest that combining ordinal learning with focal weighting improves training stability and enhances the model’s ability to handle difficult and imbalanced samples.

**Figure 10:**
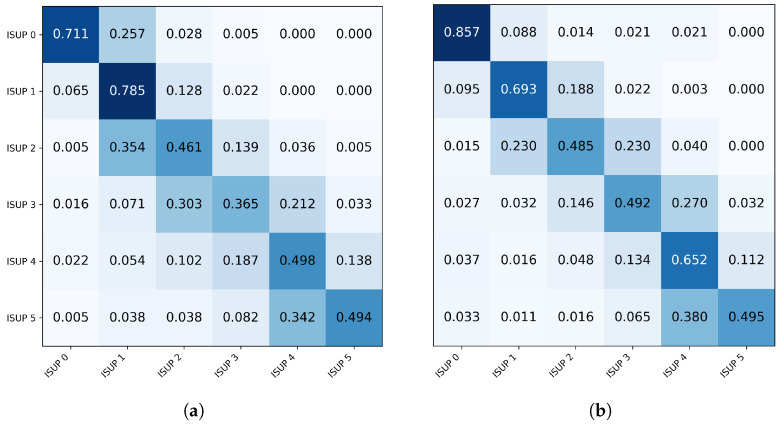
Normalized confusion matrices for models trained with ordinal loss and ordinal focal loss. The color intensity (shades of blue) represents the proportion of samples, with darker shades indicating higher values. (**a**) Ordinal; (**b**) Ordinal Focal.

The analysis of the confusion matrices presented in Figure 10 reveals consistent improvements when using the ordinal focal loss. The model trained only with ordinal loss exhibits greater dispersion across intermediate classes, particularly for ISUP class 3, whose accuracy was 0.365, with a considerable portion of samples being misclassified as ISUP 2 (0.303) and ISUP 4 (0.212). In contrast, the hybrid model shows greater concentration of predictions along the main diagonal, indicating improved discriminative capability. Relevant improvements are observed across several classes, including ISUP 0 (0.857 vs. 0.711), ISUP 3 (0.492 vs. 0.365), and ISUP 4 (0.652 vs. 0.498). In addition, a reduction in confusion between intermediate classes is observed, particularly between ISUP 2 and ISUP 3, suggesting greater separability between these histopathological grades.

Another important aspect is the improved distinction between high-grade classes. In the ordinal model, a significant proportion of ISUP 5 samples were classified as ISUP 4 (0.342). Although this confusion remains in the hybrid model (0.380), an increase in accuracy for ISUP 4 (0.652 vs. 0.498) is observed, indicating improved discrimination between higher grades.

### 3.4. Comparison Among EfficientNet Architectures

The experimental configuration defined for EfficientNet-B0 was also applied to EfficientNet-B3 and EfficientNet-B7, including the proposed hybrid loss function. The objective was to evaluate whether deeper architectures could provide additional performance gains.

The results, presented in Table 3, indicate that EfficientNet-B0 achieved the best overall performance across all evaluated metrics, including quadratic kappa, accuracy, macro F1-score, and macro precision. In particular, EfficientNet-B0 outperformed the deeper variants not only in terms of ordinal consistency (kappa) and overall correctness (accuracy), but also in balanced classification performance, as reflected by the highest F1 Macro and Precision values.

**Figure 11:**
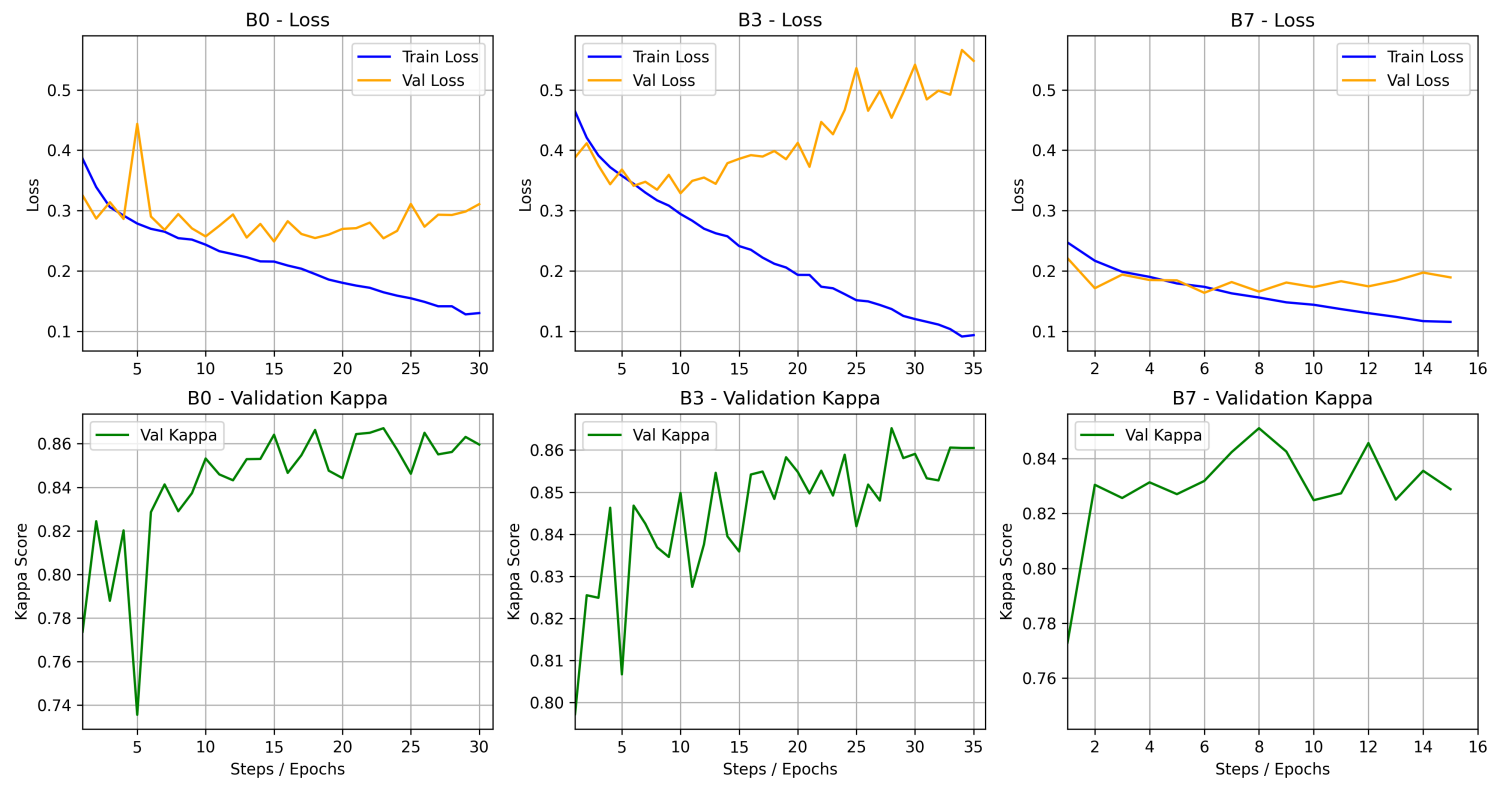
Loss and kappa curves during training and validation for different EfficientNet architectures (B0, B3, and B7).

The results indicate that the hyperparameters optimized for EfficientNet-B0 do not generalize uniformly to deeper variants of the architecture. Since the same configuration, including the learning rate and early stopping strategy, was directly applied to the B3 and B7 models, distinct training and generalization behaviors were observed. As shown in Figure 11, the B3 model exhibited clear signs of overfitting, characterized by a continuous decrease in training loss alongside a progressive increase in validation loss. Nevertheless, it achieved performance comparable to B0 in terms of kappa, suggesting that its higher representational capacity partially compensated for the lack of adequate regularization, allowing it to better fit the underlying signal despite reduced generalization. In contrast, the B7 model showed no signs of overfitting but also did not achieve superior performance. Instead, it displayed rapid convergence and early stabilization of the metrics, indicating difficulty in fully leveraging its higher capacity. This behavior suggests undertraining, where hyperparameters calibrated for a smaller architecture, particularly the learning rate and early stopping criterion, limited the optimization and prevented the model from reaching its full potential. Overall, these findings reinforce the idea that hyperparameters do not scale linearly with model depth, highlighting the need for architecture-specific tuning, especially for learning rate, regularization strategies, and the number of training epochs.

## 4. Discussion

The automated classification of ISUP grade groups faces significant challenges due to the high visual similarity between adjacent grades, the inherent subjectivity of human assessment, and the natural imbalance of clinical datasets. The results of this study demonstrate that combining an ordinally penalized loss function with noise filtering techniques provides a robust approach to overcoming these limitations.

### 4.1. Performance and Comparison with Related Work

The studies presented in Table 4 highlight different trade-offs between model complexity, task formulation, and clinical applicability. Early approaches such as [11] rely on computationally intensive pipelines that process millions of patches and multiple CNNs, limiting scalability. Similarly, refs. [12,15] operate at the patch level, simplifying the problem and ignoring global tissue context, which is essential for reliable whole-slide image (WSI) grading.

Other works adopt more complex architectures at the WSI level. For instance, ref. [8] combines convolutional and graph-based models, achieving a high internal Kappa (0.931) but with a noticeable drop in external validation (0.801), indicating sensitivity to domain shifts. Likewise, ref. [16] reports a competitive Kappa (0.881) using a multiple instance learning (MIL) ensemble; however, this result is obtained under a forced class balancing scheme applied before dataset splitting, which alters the natural data distribution. Since class imbalance is intrinsic to prostate cancer grading, this strategy may lead to overestimated performance and reduced robustness under realistic conditions. Similarly, ref. [18] achieves a Kappa of 0.884 using self-supervised learning, but relies on curated subsets, which may affect generalization.

In addition, ref. [17] incorporates a segmentation stage with DeepLabV3 followed by a Random Forest classifier, achieving a high Kappa (0.921). However, this approach depends on pixel-level annotations, limiting scalability. On the other hand, ref. [14] reports very high accuracy (up to 0.961), but addresses a simplified task (binary or reduced class setting), making comparisons with full ISUP grading less meaningful.

In contrast, the proposed method achieves a Kappa of 0.857 with a relatively simple and computationally efficient EfficientNet-B0 architecture. It addresses the full ISUP grading problem at the WSI level without relying on pixel-level annotations, artificial balancing, or task simplification. The use of Ordinal Focal Loss encourages errors between adjacent classes, thereby improving prediction consistency. Although the proposed model reports lower accuracy than some simplified approaches, it provides a more realistic and methodologically rigorous evaluation, offering a better balance between performance and robustness within the studied dataset.

### 4.2. Impact of the Ordinal Loss Function and Noise Filtering

The transition from the BCE loss function to the hybrid formulation (ordinal + focal loss) produced the largest improvement in model performance. In this context, distinguishing between adjacent ISUP grades represents less severe discrepancies than confusion between distant classes (e.g., ISUP 1 and ISUP 5). The ordinal loss encourages the model to respect this hierarchical relationship between classes. Furthermore, the entropy-based filtering strategy reinforced the importance of training data quality. Removing samples with high uncertainty reduced the influence of potentially noisy labels and improved model performance, without requiring dataset expansion.

### 4.3. Computational Efficiency and Architecture Selection

When comparing architectures from the EfficientNet family, increasing model complexity did not result in improved performance; in fact, a slight degradation was observed for deeper variants (as shown in Table 3). EfficientNet-B0 achieved the best balance between predictive performance and statistical stability. These results suggest that more complex architectures (such as B3 and B7) may be more susceptible to overfitting in this context or may require more refined training adjustments to reach their full potential. For practical implementations in pathology laboratories, selecting EfficientNet-B0 is advantageous due to its lower computational cost and shorter inference time.

### 4.4. Limitations and Future Work

Despite the promising results, an important limitation of this study is the absence of external validation on data from independent institutions. Although the PANDA dataset includes samples from two different centers, both are incorporated into the training process, which does not fully reflect real-world deployment scenarios with unseen acquisition conditions. Previous studies have shown that models trained on PANDA may experience a significant performance drop under external validation (e.g., a decrease of approximately 0.13 in kappa reported in [8]). Therefore, the generalization capability of the proposed model to different clinical environments remains uncertain, and the results presented here should be interpreted as internal performance.

Future work should prioritize evaluation on external datasets to assess robustness and support clinical applicability. Additionally, transformer-based architectures and ensemble strategies may be explored to further improve performance and stability.

## 5. Conclusions

In this study, a robust methodology was developed for the automatic classification of ISUP grades in prostate histopathological images, addressing relevant challenges, including class imbalance, label noise, and the ordinal nature of tumor progression. The introduction of a hybrid loss function, combining ordinal regression and focal loss, improved model performance by explicitly capturing the hierarchical relationship among malignancy grades. The combination of this strategy with entropy-based noise filtering proved to be highly effective. Overall, the proposed approach increased the $\kappa_{quad}$ index from 0.826 to 0.857 (approximately 3%) and the accuracy from 0.592 to 0.669 (approximately 7%) compared to the baseline.

By aligning error penalization with the ordinal structure of the problem and emphasizing data quality, the method establishes a solid foundation for automated analysis of histopathological images. While the results demonstrate improved performance within the studied dataset, they should be interpreted as internal validation, and further evaluation on independent datasets is required to assess generalization.

## Figures and Tables

**Table 1:** Evaluated hyperparameters and selected configuration for the experiments.

Parameter	Evaluated Values	Selected Value
Learning rate	$3\times 10^{-4}$, $3\times 10^{-3}$, $1\times 10^{-4}$	$3\times 10^{-4}$
Number of epochs	50	50
Unfrozen layers (*fine-tuning*)	100, 150	150
*Dropout*	0.4, 0.5, 0.6	0.4
Batch size	2	2
Loss function	BCE	BCE
Optimizer	Adam	Adam

**Table 2:** Incremental impact of the proposed strategies on the performance of EfficientNet-B0.

	Test Quadratic Kappa			Test Accuracy (%)			Test F1 Macro			Test Precision		
**Configuration**	**Result**	**SD**	**95% CI**	**Result**	**SD**	**95% CI**	**Result**	**SD**	**95% CI**	**Result**	**SD**	**95% CI**
BCE (baseline)	0.826	0.012	[0.806; 0.846]	0.592	0.012	[0.572; 0.612]	0.537	0.013	[0.516; 0.558]	0.551	0.013	[0.530; 0.572]
BCE + Noise Removal	0.833	0.013	[0.812; 0.853]	0.638	0.116	[0.619; 0.657]	0.572	0.013	[0.553; 0.594]	0.584	0.013	[0.563; 0.606]
Ordinal Loss	0.851	0.009	[0.833; 0.869]	0.608	0.126	[0.587; 0.628]	0.559	0.014	[0.537; 0.581]	0.585	0.013	[0.563; 0.606]
Ordinal + Focal (Hybrid)	0.857	0.011	[0.833; 0.876]	0.669	0.011	[0.635; 0.683]	0.612	0.013	[0.587; 0.636]	0.628	0.013	[0.603; 0.653]

**Table 3:** Performance comparison among EfficientNet architectures using the proposed experimental configuration.

	Test Quadratic Kappa			Test Accuracy (%)			Test F1 Macro			Test Precision		
**Architecture**	**Result**	**SD**	**95% CI**	**Result**	**SD**	**95% CI**	**Result**	**SD**	**95% CI**	**Result**	**SD**	**95% CI**
EfficientNet-B0	0.857	0.011	[0.833; 0.876]	0.669	0.011	[0.635; 0.683]	0.612	0.013	[0.587; 0.636]	0.628	0.013	[0.603; 0.653]
EfficientNet-B3	0.849	0.013	[0.824; 0.870]	0.661	0.014	[0.634; 0.685]	0.555	0.013	[0.532; 0.576]	0.605	0.012	[0.585; 0.624]
EfficientNet-B7	0.842	0.015	[0.815; 0.866]	0.654	0.016	[0.628; 0.678]	0.556	0.013	[0.530; 0.579]	0.561	0.013	[0.536; 0.586]

**Table 4:** Comparison between the proposed method and recent studies.

Study	Dataset	Model	Level	Task	Acc.	Kappa
[11]	Radboud	Pyramidal CNN	Patch → WSI	Gleason/GG	0.773	-
[15]	PANDA	ViT	Patch	Gleason	0.800	-
[8]	PANDA	ResNet50 + GCN	WSI	ISUP (6)	-	0.931 (Int.)/0.801 (Ext.)
[14]	DiagSet	EffNet-B4 + ECA	Patch	Binary/4 classes	0.961/0.948	-
[12]	SICAPv2	VGG19	Patch	Gleason	0.780	-
[16]	PANDA	EffNet-B1 + MIL + Ensemble	WSI	ISUP (6)	-	0.881
[17]	PANDA	EffNet-B0 + DeepLabV3 + RF	WSI + Seg	ISUP (6)	-	0.921
[18]	PANDA/SICAPv	TSOR	WSI	ISUP (6)	-	0.884
Proposed (Base)	PANDA	EffNet-B0 + Ordinal Focal Loss	WSI	ISUP (6)	0.669	0.857

## Data Availability

Publicly available datasets were analyzed in this study. The data presented in this study are openly available in the PANDA Challenge dataset at https://www.kaggle.com/c/prostate-cancer-grade-assessment (accessed on 13 March 2026). All processed results and model configurations used for analysis are available from the corresponding author upon reasonable request.

## Abbreviations

The following abbreviations are used in this manuscript:

ISUP	International Society of Urological Pathology
WSI	Whole Slide Image
TSOR	Task-Specific Ordinal Regression
CNN	Convolutional Neural Network
GCN	Graph Convolutional Network
AI	Artificial Intelligence
BCE	Binary Cross Entropy
GG	Grade Group
PANDA	Prostate Cancer Grade Assessment
ViT	Vision Transformers

## References

[1] Siegel R.L., Giaquinto A.N., Jemal A. (2024). Cancer statistics, 2024. CA Cancer J. Clin..

[2] Bray F., Laversanne M., Sung H., Ferlay J., Siegel R.L., Soerjomataram I., Jemal A. (2024). Global cancer statistics 2022: GLOBOCAN estimates of incidence and mortality worldwide for 36 cancers in 185 countries. CA Cancer J. Clin..

[3] Epstein J.I., Egevad L., Amin M.B., Delahunt B., Srigley J.R., Humphrey P.A., Grading Committee (2016). The 2014 International Society of Urological Pathology (ISUP) Consensus Conference on Gleason Grading of Prostatic Carcinoma: Definition of Grading Patterns and Proposal for a New Grading System. Am. J. Surg. Pathol..

[4] Bulten W., Pinckaers H., van Boven H., Vink R., de Bel T., van Ginneken B., van der Laak J., Hulsbergen-van de Kaa C., Litjens G. (2020). Automated deep-learning system for Gleason grading of prostate cancer using biopsies: A diagnostic study. Lancet Oncol..

[5] Rusliyawati R., Sutyarso S., Wantoro A. (2022). Expert System Research for Prostate Cancer: A Literature Review. Comput. Biol. Med..

[6] Campanella G., Hanna M.G., Geneslaw L., Miraflor A., Silva V.W.K., Busam K.J., Brogi E., Reuter V.E., Klimstra D.S., Fuchs T.J. (2019). Clinical-grade computational pathology using weakly supervised deep learning on whole slide images. Nat. Med..

[7] Araújo F.H., Silva R.R., Medeiros F.N., Neto J.F.R., Oliveira P.H.C., Bianchi A.G.C., Ushizima D. (2021). Active contours for overlapping cervical cell segmentation. Int. J. Biomed. Eng. Technol..

[8] Xiang J., Wang X., Wang X., Zhang J., Yang S., Yang W., Han X., Liu Y. (2023). Automatic diagnosis and grading of Prostate Cancer with weakly supervised learning on whole slide images. Comput. Biol. Med..

[9] Jung M., Jin M.-S., Kim C., Lee C., Nikas I.P., Park J.H., Ryu H.S. (2022). Artificial intelligence system shows performance at the level of uropathologists for the detection and grading of prostate cancer in core needle biopsy: An independent external validation study. Mod. Pathol..

[10] Liu B., Wang Y., Weitz P., Grönberg H., Eklund M., Rantalainen M. (2022). Using deep learning to detect patients at risk for prostate cancer despite benign biopsies. iScience.

[11] Hammouda K., Khalifa F., Ghazal M., Darwish H.E., Yousaf J., El-Baz A. (2022). A Pyramidal CNN-Based Gleason Grading System Using Digitized Prostate Biopsy Specimens. Proc. Int. Conf. Pattern Recognit. (ICPR).

[12] Kosoko I., Garg A., Jain S., Hewage P. (2024). Leveraging deep learning and explainable ai for diagnosis of prostate cancer. Proceedings of the International Conference on Applied Artificial Intelligence in Medical Imaging.

[13] Afifi S.M., Kaur R., GholamHosseini H., Mousa Y., Menissy B., Taha R., Baig M., Ullah E. (2025). Prostate Cancer Detection Using Deep Learning Models on Histopathological Image Slides: An Experimental Analysis. Proc. Int. Conf. Artif. Intell. Comput. Data Sci. Appl. (ACDSA).

[14] Alici-Karaca D., Akay B. (2024). An efficient deep learning model for prostate cancer diagnosis. IEEE Access.

[15] Ikromjanov K., Sumon R.I., Bhattacharjee S., Kim H.-C., Hwang Y.-B., Choi H.-K. (2021). Whole slide image analysis and detection of prostate cancer using vision transformers. Proceedings of the International Conference on Medical Imaging and AI.

[16] Bhattacharyya R., Roy P., Banerji S., Mitra S. (2024). Efficient Grading of Prostate Cancer WSI with Deep Learning. Medical Imaging 2024: Digital and Computational Pathology.

[17] Kabir S., Sarmun R., Al Saady R.M., Vranic S., Murugappan M., Chowdhury M.E.H. (2025). Automating Prostate Cancer Grading: A Novel Deep Learning Framework for Automatic Prostate Cancer Grade Assessment using Classification and Segmentation. J. Imaging Inform. Med..

[18] Bhattacharyya R., Pal S., Mitra S. (2026). Efficient Self-Supervised Grading of Prostate Cancer Pathology. IEEE Trans. Syst. Man. Cybern. Syst..

[19] PANDA Challenge (2020). Prostate cANcer graDe Assessment Challenge.

[20] Ali I., Khan A., Khan M., Ahmad R., Ullah I. (2023). Shannon Entropy in Artificial Intelligence and Its Applications Based on Information Theory. Entropy.

[21] Lin T.-Y., Goyal P., Girshick R., He K., Dollár P. (2017). Focal loss for dense object detection. Proceedings of the IEEE International Conference on Computer Vision (ICCV).

[22] Baldi P., Brunak S., Chauvin Y., Andersen C.A., Nielsen H. (2000). Assessing the accuracy of prediction algorithms for classification: An overview. Bioinformatics.

[23] Sokolova M., Lapalme G. (2009). A systematic analysis of performance measures for classification tasks. Inf. Process. Manag..

[24] Powers D.M.W. (2011). Evaluation: From precision, recall and F-measure to ROC, informedness, markedness and correlation. J. Mach. Learn. Technol..

[25] Cohen J. (1968). Weighted kappa: Nominal scale agreement with provision for scaled disagreement or partial credit. Psychol. Bull..

[26] Efron B. (1979). Bootstrap Methods: Another Look at the Jackknife. Ann. Stat..

[27] Ozkan T.A., Eruyar A.T., Cebeci O.O., Memik O., Ozcan L., Kuskonmaz I. (2016). Interobserver variability in Gleason histological grading of prostate cancer. Scand. J. Urol..

[28] Cinar I., Cinar E. (2025). Assessment of interobserver variability in Gleason grading for prostate carcinoma. North Clin. Istanb..

